# Knowledge and Practices on Periodontal Health among Adults, Misungwi, Tanzania

**DOI:** 10.1155/2018/7189402

**Published:** 2018-11-01

**Authors:** John Michael Nyorobi, Lorna Celia Carneiro, Msafiri Nicodemus Kabulwa

**Affiliations:** ^1^Dental Unit, Misungwi District Hospital, P. O. Box 8, Misungwi, Mwanza, Tanzania; ^2^Department of Restorative Dentistry, Muhimbili University of Health and Allied Sciences, P. O. Box 65451, Dar es Salaam, Tanzania; ^3^Oral Health Section, Ministry of Health, Community Development, Gender, Elderly and Children, P. O. Box 743, Dodoma, Tanzania

## Abstract

The level of knowledge and practices on periodontal health of 388 adults in Misungwi District, Tanzania, was assessed. Analysis included frequency distributions and the chi-square test at a *p* value of 0.05. Many more participants were males (*n*=197; 50.8%) and of younger age group (*n*=215; 55.4%) and having seven years of education (*n*=257; 66.2%). Higher number of participants reported owning a mobile phone (*n*=289; 74.5%) and a radio (*n*=298; 76.8%), while few (*n*=45; 11.6%) had their houses connected to electricity. Study participants who were males (*n*=101; 51.3%), had more than seven years of schooling (*n*=67; 72.3%), who owned a mobile phone (*n*=143; 49%), owned a radio (*n*=144; 48.3%), and resided in houses having electricity (*n*=37; 82.2%) were statistically significantly more knowledgeable on periodontal health when compared to their counterparts. A statistical significant difference was seen in oral health practices conducive to periodontal health among those who were males (*n*=133; 67.0%), having more than seven years of education (*n*=78; 83.0%), owning a mobile phone (*n*=189; 65.4%), owning a radio (*n*=195; 65.4%), and residing in house having electricity (*n*=35; 77.8%). This study observed that participants who were males, owned a mobile phone, owned a radio, resided in houses having electricity, and having more than seven years of education were more knowledgeable and had more conducive oral health practices on periodontal health.

## 1. Introduction

Periodontal disease is a chronic disease that affects one or more components of the periodontium and is a growing burden to people, healthcare systems, and societies across the world [1]. It refers to the inflammatory processes that occur in the tissues surrounding the teeth in response to bacterial accumulations (dental plaque) [2]. Gingivitis and periodontitis are a continuum of the same inflammatory process [3], and while gingivitis is the presence of gingival inflammation without loss of connective tissue attachment, periodontitis is the presence of gingival inflammation at sites where there has been apical migration of the epithelial attachment onto the root surfaces accompanied by loss of connective tissue and the alveolar bone [4].

Periodontal disease has been associated with self-reported signs and symptoms-like ‘‘swollen gums,” ‘‘sore gums,” ‘‘receding gums,” ‘‘loose teeth,” ‘‘drifting teeth,” ‘‘bad breath,” and ‘‘toothache” [5]. Furthermore, severe periodontitis, which may result in tooth loss [6], is found in 5–20% of most adult populations worldwide [7].

The prevalence of periodontal disease in a population is dependent on risk characteristics like age, sex, education, geographic and environmental status, oral hygiene practices, and social characteristics [8]. In addition, dental awareness, oral health, and oral health-related behaviors have been reported to be influenced also by the socioeconomic status [9], and the link between periodontal disease and socioeconomic status has been reported [10]. Socioeconomic status associated with the health status of an individual including oral health has been assessed using various indicators such as owning a television, refrigerator, mobile phone, bicycle, car, utensils, access to utilities (electricity and water), and housing characteristics (materials used for floor, number of rooms, and quality of toilets) [11]. It has been further documented that higher income enables people to afford better housing and permit increased access to healthcare and offers the opportunity to adopt appropriate oral health practices [12].

So as to maintain oral health and prevent the occurrence of periodontal diseases in a population, it is essential that there be acquisition of adequate oral health knowledge together with instilling of appropriate self-care practices [13]. Moreover, prevention of and early intervention into periodontal disease are critical, and successful management of periodontal disease depends on the capacity of patient's oral self-care practices [14, 15]. The single most important step an individual can take to reduce plaque accumulation and the consequent risk of plaque-associated diseases, such as periodontitis, is perhaps routinely performing tooth brushing [16]. With regard to frequency of tooth brushing, a previous study demonstrated that individuals who cleaned their teeth at least twice a day exhibited less visible plaque, as compared with those who cleaned their teeth less than once a day or never [17]. However, the benefit of toothpaste in removing plaque during brushing has been controversial [18].

In Tanzania, dental plaque has been reported to be prevalent among adolescents [19] and adults [20], and it is estimated that, on average, at the age of 40 to 49 years, each Tanzanian has lost at least one tooth because of advanced periodontal disease, and after the age of 50, the problem becomes more marked [21]. To address this, an oral health policy to promote primary, secondary, and tertiary levels of oral healthcare to Tanzanians through community involvement particularly in the rural areas was developed [22].

A previous study on oral health issues for the elderly in Tanzania reported that people aged 40+ years, who live in the rural areas, are at a higher risk of destructive periodontal disease, and it was recommended that oral health education, focusing on behavior change, should be initiated from childhood [23].

Identifying the level of oral health knowledge and practices on periodontal health can assist in planning preventive strategies towards reducing periodontal disease. This study therefore aimed at determining the level of knowledge and practices on periodontal health among adults of Misungwi District in Tanzania.

## 2. Materials and Methods

Misungwi District in Mwanza Region in Tanzania has 4 divisions, 20 wards, 78 villages, and 40,364 households, with a total population of 316,687 people [24]. Using multistage stratified cluster random sampling, two of the four divisions within Misungwi District were selected. Subsequently, in each division, one ward was selected, and from each ward, two villages were chosen. From each village, ten households are grouped and referred to as ten cells, and in each village, 25 ten cells were visited. Adults present in each household within a ten cell were informed of the purpose of the study by the local administrative authority following which consent was obtained. Participants excluded from the study were those below 18 years of age, adults who were nonresidents of Misungwi, and those who were either mentally challenged or sick.

Following consent, a structured self-administered questionnaire that was translated into Kiswahili was used to gather the necessary information from the study participants. The questionnaire was divided into four sections, namely, sociodemographic information, socioeconomic status indicators, assessment of the level of knowledge, and assessment of practices on periodontal health. Pretesting to establish meaning and clarity of the questionnaire was done using a randomly selected group of forty people from one of the villages (Buhunda). Following testing, words and sentences that appeared not clear were rectified and retesting of the questionnaire was done. Reproducibility of the answers ranged between 90 and 100% indicating adequate reliability of the questionnaire.

Sociodemographic information included details on age (young adults-18–34 years and middle aged to elderly adults-35–74 years), sex (male and female), and level of education (not gone to school, seven years of education, and more than seven years of education).

Socioeconomic status indicators included information on owning a radio (yes = 1 or no = 0) or mobile phone (yes = 1 or no = 0) or having electricity in household (yes = 1 or no = 0).

Level of knowledge on periodontal health was assessed using 6 questions (knowledge on types of oral diseases, presentation of the gum disease, causes of gum disease, effects of gum disease, prevention of gum disease, and harmful effects to the gum due to smoking). Responses to the questions were itemized and coded following which incorrect responses were awarded a zero mark, and correct responses were awarded a mark of one. A count of correct responses to all questions was done, and a subject was regarded as being knowledgeable if three or more of the six questions were correctly answered and not knowledgeable if less than three of the questions were correctly answered.

Level of oral health practice on periodontal health was assessed using 5 questions, namely, use of toothpaste for brushing (yes = 1 or no = 0), time spent during tooth brushing (less than two minutes during tooth brushing, and two or more minutes during tooth brushing), frequency of tooth brushing (once a day or two or more times a day), having attended a dental health facility (yes = 1 or no = 0), and practice of smoking (yes = 1 or no = 0). Responses to the five questions were counted, and participants who scored 3 or more were considered to have practices conducive for periodontal health, while participants who scored below three were regarded as having practices not conducive for periodontal health.

Data processing was done using Statistical Package of the Social Sciences, IBM^™^, version 17.0 of 2016 (Chicago, IL, USA). Independent variables were sociodemographic details (age, sex, and level of education) and socioeconomic status indicators (owning a radio or mobile phone or having electricity), while dependent variables were questions on oral health knowledge and practices towards periodontal health. Data analysis included frequency distribution and cross tabulation. Frequency distributions of respondent's responses to the questions on knowledge and practice were generated. The chi-square test was performed to identify associations between independent and dependent variables, and a *p* value of 0.05 was used to determine the level of statistical significance.

Prior to conducting the study, a Research clearance Certificate Number CREC/039/2013 was obtained from the Catholic University of Health and Allied Sciences (CUHAS)/Bugando Medical Centre (BMC) Research Ethical Committee (CREC) and used to obtain permission from the Regional and District authorities of Misungwi.

## 3. Results

A total of 388 adults participated in this study with an age ranging from 18 to 74 years and median age of 32 ± 0.58 years. With regard to sociodemographic details, there were slightly more males (50.8%) than females and young adults under the age of 35 years (55.4%). Participants who had not gone to school were the minority (*n*=37; 9.6%), while those who had seven years of education were the majority (*n*=257; 66.2%). The remaining participants had more than seven years of education (*n*=94; 24.2%). On assessment of socioeconomic status indicators, about three-quarters (*n*=289; 74.5%) owned mobile phones and 76.8% (*n*=298) owned a radio, while only few (*n*=45; 11.6%) had their houses connected to electricity. Owning of a mobile phone and radio among those who had not gone to school was 4.4% and 7.2%, respectively. About half (*n*=190; 49.0%) of the participants could mention any oral disease, 68.6% (*n*=266) could mention signs and symptoms of periodontal diseases, and about a quarter (*n*=103; 26.5%) could mention causes of gum diseases, while 32% (*n*=124) and 29.6% (*n*=115) could mention effects and prevention of gums diseases, respectively. About a third (*n*=129; 33.2%) were aware on the harmful effects of smoking on gum diseases. Majority of the participants used toothpaste during brushing their teeth (*n*=321; 82.7%) and reported to spend two minutes or more during tooth brushing (*n*=336; 86.6%), while less than half (*n*=180; 46.4%) brushed their teeth two or more times a day. A little more than a third (*n*=154; 39.7%) had ever attended a dental health facility, and a minority (*n*=59; 15.2%) reported that they smoke (Table 1).

Table 2 shows the distribution of the participant's level of knowledge on periodontal health by sociodemographic and socioeconomic variables. Compared to females, male participants were more knowledgeable (*n*=101; 51.3%) on periodontal health, and the difference was statistically significant (*p* value = 0.018). With regard to age, there was no statistical significant difference (*p* value = 0.082) between level of knowledge on periodontal health of young adults (*n*=106; 49.3%) when compared to middle aged to elderly participants (*n*=70; 40.5%). Study participants who had more than seven years of schooling were statistically significantly more knowledgeable (*n*=68; 72.3%) on periodontal health when compared to their counterparts (*p* value = 0.0001). Participants residing in houses having electricity (*n*=37; 82.2%) had a statistically significantly higher (*p* value = 0.0001) level of knowledge on periodontal health when compared to participants residing in houses not connected to electricity (*n*=139; 40.5%). Participants owning a mobile phone (*n*=143; 49.5%) were statistically significantly more knowledgeable on periodontal health than those who did not own mobile phones (*n*=33; 33.3%) with a *p* value of 0.005. Compared to those who did not own a radio (*n*=32; 35.6%), the ones who owned it (*n*=144; 48.3%) were statistically significantly more knowledgeable on periodontal health (*p* value = 0.033).

Table 3 shows the distribution of the participant's level of oral health practices on periodontal health by sociodemographic and socioeconomic variables. Proportionately, there were more male (*n*=133; 67.0%) compared to female (*n*=108; 56.5%) participants who had conducive oral health practices (*p*=0.034). Participants aged 18–34 years (*n*=132; 61.4%) and those aged 35 years and above (*n*=108; 62.4%) showed no statistical significant differences in their oral health practices (*p*=0.835). A high statistical significant difference (*p* value = 0.0001) was seen in oral health practices conducive to periodontal health among participants who did not go to school (*n*=18; 48.6%), with seven years of education (*n*=144; 56.0%), and those with more than seven years of education (*n*=78; 83.0%). Practices conducive to oral health were statistically significantly different (*p*=0.014) among participants who owned (*n*=189; 65.4%) or did not own a cell phone (*n*=51; 51.5%). Owning a radio (*p*=0.008) or living in houses with electricity (*p*=0.019) was seen to be a contributing factor towards practices conducive to oral health.

## 4. Discussion

This study assessed the level of knowledge and practices on periodontal health among adults of Misungwi District in Tanzania using a representative sample of selected clusters of households. These findings are adequate to give some insights into the level of knowledge and practices on periodontal health of adults in Misungwi, Tanzania, and provide baseline data for planning purposes.

With regard to sociodemographic findings of this study, majority of participants were males (50.8%) and of the younger age group (55.4%) and having seven years of education (66.2%). Studies conducted in Tanzania [25] and Brazil [26] also reported similar findings. Being a developing country, it is expected that the number of young adults compared to older adults in Tanzania will be higher. Furthermore, the higher number of participants with seven years of education could be a reflection of the government efforts to make primary school free and accessible to all [27]. The higher number of males in this study is contrary to findings reported in another study conducted among adults in Tanzania [28]. This difference could be attributed to the differences in study design and sample size.

When we assessed the socioeconomic status as reflected in the 2012 Tanzanian Census (19), the prevalence of the mobile phone and radio ownership was high at 74.5 and 76.8%, respectively, while the percentage of households with a connection to electricity was low at 11.6%.

It is speculated that income generated from either employment, farming, or other business together with reasonable purchasing costs could have enabled the owning of a mobile phone or radio by participants. Furthermore, electricity to the majority of the population in Tanzania is still a challenge and similar to findings reported in this study, and it has been reported that only few households in Tanzania currently have electricity [29]. It is speculated that the few participants who had electricity in their houses had their houses close to an already installed electrical system and hence were able to obtain electricity for a nominal fee.

Assessment of the study participant's knowledge on periodontal health showed that many more could mention signs and symptoms of periodontal diseases (68.6%) compared to mentioning of any oral disease (49.0%), causes (26.5%), effects (32%), and prevention (29.6%), while about a third (33.2%) were aware on the harmful effects of smoking on periodontal disease. These findings are comparable to a study conducted in Finland [30]. Contrastingly in the United Kingdom [31] and Kuwait [32], a higher proportion of study participants were knowledgeable on effects of smoking rather than on signs and symptoms of periodontal diseases. Personal experience and observation by participants could have contributed to the reported higher level of knowledge on signs and symptoms of periodontal disease in this study.

Assessed oral health practices on periodontal health by participants reported that the majority (82.7%) used toothpaste and spend two minutes or more during tooth brushing (86.6%) while less than half (46.4%) brushed their teeth two or more times a day. The use of toothpaste by majority was also reported by Kikwilu et al. [33]. Similar to results of this study, the general consensus amongst oral healthcare professionals is that individuals should spend at least 2 minutes brushing their teeth with an effective technique at least twice a day [34]. The proportion of participants (39.7%) who reported the practice of having attended a dental health facility in the present study was similar to other studies done in Tanzania [25, 35] and different to reports from developed countries like Germany [36] and USA [37]. Furthermore, smoking practiced by a few (15.2%) participants in this study was similar to results of the standardized estimate of smoking reported in Tanzania by the World Health Organization [38]. The reported oral health practices could be attributed to marketing strategies employed to ensure that toothpaste is readily available at a reasonable cost, lack of availability of a health facility or not having the means to access the service, and personal or social choices made by few who smoke.

Study participants who were males (51.3%), had more than seven years of education (72.8%), residing in houses having electricity (82.2%), and who owned a radio (48.3%) were statistically significantly more knowledgeable on periodontal health when compared to their counterparts.

Tanzanian males were significantly more knowledgeable about periodontal disease than their female counterparts, which is in agreement with the findings of a previous study conducted in India (34) but in contrast to the results of a Nigerian study (35).

The significant statistical difference in oral health knowledge observed between male and female may be due to inequalities in the opportunity to education within communities which favors males more than females. Furthermore, inequalities in educational opportunities for females could have been contributed to by tradition and culture which favored early marriage of females and early drop out from school due to getting pregnant. It is anticipated that this difference will be decreased in the coming years as the government has made efforts to make primary school free and accessible to all [27].

A highly statistical significant difference (*p* value = 0.0001) was seen in oral health practices conducive to periodontal health between participants who had not gone to school, with seven years of education, and those with more than seven years of education (83.0%). Other studies [39, 40] have also reported the level of education as a strong determinant of oral hygiene practices. Furthermore, the level of education was suggested as a potential risk indicator for oral health practices [41, 42]. Being educated probably gives one the foundation and basis of understanding and the ability to make decisions with regard to practices conducive to oral health.

Owning a radio (*p*=0.008), a mobile phone (*p*=0.014), or living in houses with electricity (*p*=0.019) was seen to be a contributing factor towards practices conducive to oral health. The observed findings could have been contributed to by participants having adequate knowledge on oral health that heightened the observed practices. In addition, the observed oral healthcare practices could have been enabled by the interlinking of the various socioeconomic variables. Furthermore, ownership of the mobile phones or radios can be used in future as a means of assessment of knowledge and practices on oral health within a community.

## 5. Conclusions

Participants in this study who were males, owned a mobile phone, owned a radio, resided in houses having electricity, and having more than seven years of education were more knowledgeable and had more conducive oral health practices on periodontal health. These findings can be used to develop strategies for oral health promotion and control of periodontal diseases in the country.

## Figures and Tables

**Table 1:** Frequency distribution of the study participants' oral health knowledge and oral health practices on periodontal health by sociodemographic and socioeconomic variables (*n*=388).

	Number (*n*)	Percentage (%)
Sociodemographic variables		
Sex		
Male	197	50.8
Female	191	49.2
Age group		
18–34	215	55.4
35+	173	44.6
Level of education		
Not gone to school	37	9.6
Seven years of education	257	66.2
More than seven years of education	94	24.2

Socioeconomic variables		
Owning a cell phone		
Yes	289	74.5
No	99	25.5
Owning a radio		
Yes	298	76.8
No	90	23.2
House having electricity		
Yes	45	11.6
No	343	88.4

Oral health knowledge		
Can they mention types of oral diseases?		
Yes	190	49.0
No	198	51.0
Can they mention signs and symptoms of periodontal diseases?		
Yes	266	68.6
No	122	31.4
Can they mention causes of gum diseases?		
Yes	103	26.5
No	285	73.5
Can they mention effects of gum diseases?		
Yes	124	32.0
No	264	68.0
Can they mention prevention of gum of diseases?		
Yes	115	29.6
No	273	70.4
Awareness on harmful effects of smoking on gums		
Yes	129	33.2
No	259	66.8

Oral health practice		
Use of toothpaste during teeth brushing		
Yes	321	82.7
No	67	17.3
Time spent during tooth brushing		
Less than two minutes	52	13.4
Two minutes or more	336	86.6
Frequency of tooth brushing		
Less than twice a day	208	53.6
Twice or more a day	180	46.4
Ever attended dental health facility?		
Yes	154	39.7
No	234	60.3
Do you smoke?		
Yes	59	15.2
No	329	84.8

**Table 2:** Distribution of the participant's level of knowledge on periodontal health by sociodemographic and socioeconomic variables.

		Level of oral health knowledge		*p* value
		Not knowledgeable	Knowledgeable	
Sociodemographic variables				
Sex	Male	96 (48.7%)	101 (51.3%)	0.018
	Female	116 (60.7%)	75 (39.3%)	
Age (years)	18–34	109 (50.7%)	106 (49.3%)	0.082
	35+	103 (59.3%)	70 (40.5%)	
Level of education	Not gone to school	30 (81.1%)	7 (18.9%)	0.0001
	Seven years of education	156 (60.7%)	101 (39.3%)	
	More than seven years of education	26 (27.7%)	68 (72.3%)	

Socioeconomic variables				
Owning a cell phone	Yes	146 (50.5%)	143 (49.5%)	0.005
	No	66 (66.7%)	33 (33.3%)	
Owning a radio	Yes	154 (51.7%)	144 (48.3%)	0.033
	No	58 (64.4%)	32 (35.6%)	
Houses having electricity	No	204 (59.5%)	139 (40.5%)	0.0001
	Yes	8 (17.8%)	37 (82.2%)	

**Table 3:** Distribution of the participant's level of oral health practices on periodontal health by sociodemographic and socioeconomic variables.

		Level of oral health practices		*p* value
		Not acceptable	Acceptable	
Sociodemographic variables				
Sex	Male	65 (33.0%)	132 (67.0%)	0.034
	Female	83 (43.5%)	108 (56.5%)	
Age (years)	18–34	83 (38.6%)	132 (61.4%)	0.835
	35+	65 (37.6%)	108 (62.4%)	
Level of education	Not gone to school	19 (51.4%)	18 (48.6%)	0.001
	Seven years of education	113 (44.0%)	144 (56.0%)	
	More than seven years of education	16 (17.0%)	78 (83.0%)	

Socioeconomic variables				
Owning a cell phone	Yes	100 (34.6%)	189 (65.4%)	0.014
	No	48 (48.5%)	51 (51.5%)	
Owning a radio	Yes	103 (34.6%)	195 (65.4%)	0.008
	No	45 (50.0%)	45 (50.0%)	
House having electricity	Yes	10 (22.2%)	35 (77.8%)	0.019
	No	138 (40.2%)	205 (59.8%)	

## Data Availability

Data will be made available on request by contacting the corresponding author.
